# Continental-Scale Footprint of Balancing and Positive Selection in a Small Rodent (*Microtus arvalis*)

**DOI:** 10.1371/journal.pone.0112332

**Published:** 2014-11-10

**Authors:** Martin C. Fischer, Matthieu Foll, Gerald Heckel, Laurent Excoffier

**Affiliations:** 1 Computational and Molecular Population Genetics (CMPG), Institute of Ecology and Evolution, University of Bern, Bern, Switzerland; 2 Institute of Integrative Biology, ETH Zürich, Zürich, Switzerland; 3 School of Life Sciences, École Polytechnique Fédérale de Lausanne (EPFL), Lausanne, Switzerland; 4 Swiss Institute of Bioinformatics, Lausanne, Switzerland; North Carolina State University, United States of America

## Abstract

Genetic adaptation to different environmental conditions is expected to lead to large differences between populations at selected loci, thus providing a signature of positive selection. Whereas balancing selection can maintain polymorphisms over long evolutionary periods and even geographic scale, thus leads to low levels of divergence between populations at selected loci. However, little is known about the relative importance of these two selective forces in shaping genomic diversity, partly due to difficulties in recognizing balancing selection in species showing low levels of differentiation. Here we address this problem by studying genomic diversity in the European common vole (*Microtus arvalis*) presenting high levels of differentiation between populations (average *F*_ST_ = 0.31). We studied 3,839 Amplified Fragment Length Polymorphism (AFLP) markers genotyped in 444 individuals from 21 populations distributed across the European continent and hence over different environmental conditions. Our statistical approach to detect markers under selection is based on a Bayesian method specifically developed for AFLP markers, which treats AFLPs as a nearly codominant marker system, and therefore has increased power to detect selection. The high number of screened populations allowed us to detect the signature of balancing selection across a large geographic area. We detected 33 markers potentially under balancing selection, hence strong evidence of stabilizing selection in 21 populations across Europe. However, our analyses identified four-times more markers (138) being under positive selection, and geographical patterns suggest that some of these markers are probably associated with alpine regions, which seem to have environmental conditions that favour adaptation. We conclude that despite favourable conditions in this study for the detection of balancing selection, this evolutionary force seems to play a relatively minor role in shaping the genomic diversity of the common vole, which is more influenced by positive selection and neutral processes like drift and demographic history.

## Introduction

Despite nearly six decades of genetic investigations, it remains unclear for most organisms to which extent the demographic history of populations, genetic drift or selection influences the pattern of genetic diversity of a species. Historically, the observation that many genes are genetically polymorphic within population was first explained by a selective advantage of heterozygotes [1]. This explanation was challenged by Kimura's neutral [2], [3] and nearly neutral [4] theory of molecular evolution, which provided a competing explanation for the high frequency of genetic polymorphism. Nowadays it is generally accepted that a majority of the genetic variations evolved nearly neutrally, but that natural selection plays a decisive role in evolution and leaves footprints in the genome. Natural selection acts in at least three forms, which are positive, purifying and balancing selection. Positive selection can lower genetic diversity locally but increase it globally, to a level depending on the spatial and environmental heterogeneity [5]–[7]. Balancing selection maintains genetic variation within populations [8] and leads to generally low levels of differentiation between populations, even though it can contribute to increase population differentiation if selective pressures are spatially heterogeneous [5]. Finally, purifying selection generally decrease levels of genetic diversity, even though strong background selection can promote increased difference between populations by lowering their effective size [9]. In the past, balancing selection played an important role in evolutionary genetics in explaining the high level of genomic polymorphism observed among species or populations [8], [10]. However, the effect of selection can be multifarious and the impact of each is still under debate [11], especially for balancing selection.

At least in humans a number of common genetic diseases have been proposed to be maintained in populations as a result of balancing selection, e.g. sickle-cell anaemia [12], [13], glucose-6-phosphate dehydrogenase deficiency [14], thalassemia [15] and cystic fibrosis [16]. Other examples are the ABO blood group [17], polymorphisms of beta-globin [18], the major histocompatibility complex (MHC; [19]) including the human HLA-G promoter [20], CCR5 in humans [21], the complementary sex determination locus in bees [22], response to pathogens [23], high diversity genes in *Arabidopsis* [24] or self-incompatibility and nuclear-cytoplasmic gynodioecy in plants (see e.g. [25]). However, all these examples were identified by a candidate gene approach and not genome-wide scans. Hence they do not give any information about the importance of balancing selection in shaping genomic diversity. In this context, there are only few genome-wide studies of balancing selection in humans [26], [27] or sticklebacks [28] and these studies remain inconclusive about the importance of balancing selection in shaping and maintaining genetic diversity, potentially due to methodological limitations (see below). Compared to balancing selection, the occurrence and influence of positive selection on an organism's genetic variation is much less questioned, as positive selection should allow the spread of advantageous traits and play a central role in the evolution of species (see e.g. [29], [30]).

The prevalence of balancing selection is still highly debated, mainly due to missing evidence in organisms other than humans, but also because methods developed specifically to detect balancing selection are still few (see e.g. [27], [31]–[34]). Moreover, the classical detection of balancing selection based on levels of differentiation between populations is difficult in organisms with low levels of differentiation (see [35]) like humans or *Drosophila* [36], [37] and a decent number of populations need to be investigated to have the statistical power to detect balancing selection [35].

**Figure 1:**
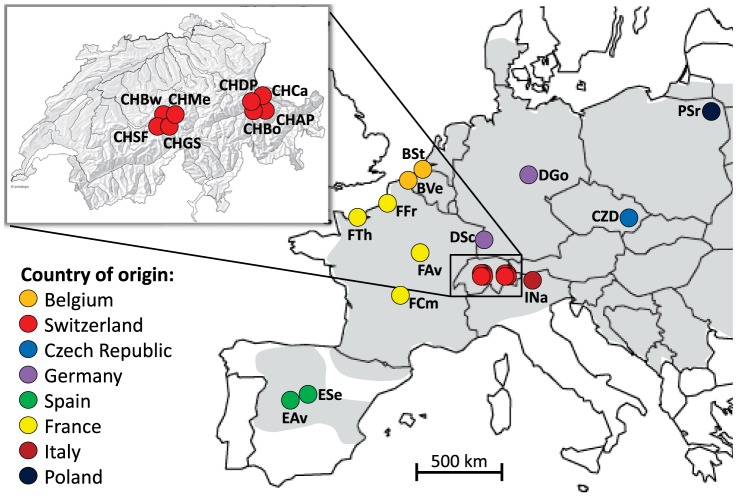
Geographic location of the 21 *Microtus arvalis* populations analyzed. The grey area corresponds to the European distribution of the species (after [98]). See Table 1 for sample abbreviations.

In order to better detect signals of balancing selection, we focused in this study on an organism showing particularly high levels of differentiation, which is the common vole (*Microtus arvalis*). This species has a very wide distribution in Europe, and it is found in most open grassland and farmland habitats up to 2,000 m altitude [38]–[40]. It ranges from the Atlantic coast of France to Central Russia, as well as from the Orkney archipelago in the North to the Mediterranean coast in Spain (Figure 1). In previous studies it has been shown that the vole populations have an overall high levels of differentiation for both mtDNA (*F*_ST_ = 0.7) and nuclear markers (STR, *F*_ST_ = 0.17) [41]–[43]. The widespread distribution of this species in different habitats and environments, and its peculiar pattern of genetic diversity makes it particularly suitable for the detection of markers with high or low levels of differentiation, and by extension for the determination of the respective roles of positive or balancing selection over a large geographic scale.

The aim of this study is to detect selective patterns in populations across the European mainland to disentangle the importance of balancing and positive selection in shaping the genetic diversity observed in the distribution range of the common vole. However, a major challenge in identifying genomic regions under selection is to separate the footprint of selection from that of population history and demography (e.g. [10], [44], [45]). Hence examining a large number of loci scattered throughout the genome is an effective way to tell apart the effect of selection from the confounding effects of population history and demography [10], [46], [47]. Cavalli-Sforza [48] and Lewontin and Krakauer [49] proposed that genetic drift and gene flow should affect all loci similarly, leading to some overall degree of differentiation between populations, but that selected loci would deviate significantly from this distribution. Indeed, positive selection acting on a given locus should increase population differentiation (and lead to high *F_ST_*) whereas balancing selection should reduce it and lead to low *F_ST_* (see e.g. [40], [47], [50], [51]).

For non-model organisms Amplified Fragment Length Polymorphisms (AFLPs) allow the screening of thousands of randomly distributed loci in a genome [52], [53]. To detect AFLP outliers, we used a recently developed extension of the Bayesian *F*_ST_-based approach [35], [54] based on the *F*-model [55]. BayeScan 2.1 [40] provides estimates of allele frequencies and *F*-statistics from AFLPs by incorporating for each individual the band intensity of a marker instead of simply using presence-absence patterns [56], [57]. This procedure implicitly allows one to distinguish between homo- and heterozygotes, and significantly improves the detection of selection with AFLP markers, which nearly reach the power obtained with single nucleotide polymorphism (SNP) data for which individual genotypes are known [40].

## Materials and Methods

### Sample and DNA extraction

We analysed 21 vole populations across most of the distribution range of *M. arvalis* in Europe, with a total of 444 individuals (see Figure 1 and Table 1). The populations were spread over 2,500 km from Spain (EAv) to Poland (PSr), and over a 750 km latitudinal gradient from Belgium (BSt) to Italy (INa).

**Table 1:** Location and properties of 444 *M. arvalis* samples from 21 populations across Europe.

ID	Country	Location	N	Latitude	Longitude	*F*_ST_ (CI)	*F_IS_* (CI)	Variable AFLPs %	AFLP diversity
BSt	Belgium	Stalhille	21	51°13′N	3°05′E	0.304 (0.292–0.318)	0.0010 (0.0000–0.0029)	30.2	0.091
BVe	Belgium	Veurne	21	51°03′N	2°40′E	0.345 (0.331–0.359)	0.0008 (0.0000–0.0024)	25.3	0.078
CZD	Czech Republic	Drnholec	21	48°53′N	16°27′E	0.162 (0.151–0.172)	0.0431 (0.0288–0.0576)	46.3	0.132
FAv	France	Avallon	20	47°29′N	3°54′E	0.248 (0.236–0.261)	0.0008 (0.0000–0.0023)	37.3	0.115
FCm	France	Clérmont-Ferrand	21	45°47′N	3°05′E	0.262 (0.250–0.275)	0.0006 (0.0000–0.0018)	38.1	0.116
FFr	France	Fressenneville	22	50°05′N	1°34′E	0.302 (0.289–0.315)	0.0020 (0.0001–0.0058)	30.8	0.090
FTh	France	Thaon	21	49°16′N	0°26′W	0.294 (0.281–0.306)	0.0058 (0.0004–0.0137)	35.4	0.108
DGo	Germany	Gotha	20	50°57′N	10°42′E	0.224 (0.212–0.235)	0.0238 (0.0118–0.0382)	37.6	0.113
DSc	Germany	Schiltach	20	48°17′N	8°21′E	0.237 (0.225–0.250)	0.0007 (0.0000–0.0022)	33.8	0.107
INa	Italy	Naturno	21	46°39′N	11°01′E	0.349 (0.335–0.363)	0.0030 (0.0002–0.0083)	31.6	0.096
PSr	Poland	Srodkowy	21	53°35′N	22°47′E	0.297 (0.283–0.309)	0.0055 (0.0000–0.0136)	34.2	0.108
EAv	Spain	Avila	21	40°42′N	4°48′W	0.426 (0.411–0.440)	0.0009 (0.0000–0.0026)	27.8	0.084
ESe	Spain	Segovia	21	40°54′N	4°06′W	0.442 (0.429–0.457)	0.0006 (0.0000–0.0019)	26.1	0.078
CHAP^1^	Switzerland	Alp di Plaun	20	46°47′N	9°29′E	0.224 (0.212–0.236)	0.0129 (0.0001–0.0240)	39.3	0.123
CHBo^1^	Switzerland	Bonaduz	22	46°48′N	9°24′E	0.297 (0.285–0.310)	0.0050 (0.0000–0.0125)	32.3	0.098
CHBw^2^	Switzerland	Brienzwiler	21	46°45′N	8°07′E	0.328 (0.315–0.343)	0.0031 (0.0000–0.0085)	27.7	0.082
CHCa^1^	Switzerland	Calandahütte	22	46°53′N	9°29′E	0.257 (0.245–0.269)	0.0124 (0.0003–0.0232)	35.5	0.105
CHDP^1^	Switzerland	Domat/Ems	22	46°50′N	9°26′E	0.278 (0.265–0.290)	0.0061 (0.0000–0.0145)	32.6	0.097
CHGS^2^	Switzerland	Grosse Scheidegg	22	46°40′N	8°06′E	0.386 (0.372–0.400)	0.0011 (0.0000–0.0034)	22.6	0.068
CHMe^2^	Switzerland	Meiringen	22	46°43′′N	8°11′E	0.411 (0.396–0.424)	0.0007 (0.0000–0.0021)	19.9	0.061
CHSF^2^	Switzerland	Schreck-Feld	22	46°40′N	8°04′E	0.354 (0.341–0.368)	0.0019 (0.0001–0.0055)	25.1	0.076

We report the population ID, sampling site (country and location), number of individuals per population (N), coordinates, estimates of population specific *F*_ST_ and *F*_IS_ with 95% credibility intervals (CI), proportion of variable AFLP markers and AFLP marker diversity for each population.

1 AFLPs of six primer combinations have been published in Foll *et al.* [56].

2 AFLPs of 21 primer combinations have been published in Fischer et al. [40].

The samples for this study were obtained by strictly following the legislation on animal protection and experimentation of Switzerland and the other European countries involved. *Microtus arvalis* is not specifically protected by Swiss laws on animal protection (Tierschutzgesetz from December 16 2005) and hunting legislation (Verordnung zum Jagdschutzgesetz, February 29 1988) because of its role as an agricultural pest and general abundance. The use of snap traps for sampling *M. arvalis* is not a stress-inducing animal experiment (Schweregrad 0; Art. 137ff Swiss federal regulations on animal experimentation). However, Swiss samples analyzed in this study (some of them also covered in earlier studies; [40]–[43], [56], [58]–[62]) were obtained also under animal experimentation permits No. 55/02; 107/05; BE08/10; BE90/10 issued by the cantonal veterinary office of Bern according to federal law after ethical approval by the Bernese cantonal commission on animal experimentation. Additional samples were obtained from the researcher network on rodent-borne pathogens based at the German Federal Research Institute for Animal Health (FLI; http://www.fli.bund.de; GH is one of the coordinators) [63]–[65] and its international partners in the European projects EDEN and EDENext on biology and control of vector-borne infections in Europe (http://www.edenext.eu). Sample acquisition followed strictly the legislation of the relevant countries after approval by the according animal protection and ethics committees as required by the European Commission Seventh Framework Programme (FP7; http://cordis.europa.eu/fp7/home_en.html) [66], [67].

Total genomic DNA was extracted from foot, tail or liver tissue stored in absolute ethanol and later deep-frozen using a standard phenol-chloroform protocol [68]. The quality and quantity of the DNA was determined on 0.8% agarose gels and with a spectrophotometer (NanoDrop ND-1000 Spectrophotometer, NanoDrop Technologies, Inc., Wilmington, USA). The DNA concentration was standardized to 100 ng DNA/µL for all individuals to ensure similar PCR yield across samples [40].

### AFLP analyses

AFLP analyses were performed according to standard protocols as established by Vos et al. [52] and modified by Fink et al. [69]. Selective amplifications were performed using 21 primer combinations (Table S1). These primer combinations were then named according to the last two selective bases of each primer, e.g. the combination E01-AAC/M02-cag is referred to as *ACag*. Special care was taken to guarantee the reproducibility of AFLP marker analyses: a liquid-handling robot (Microlab STAR, Hamilton Bonaduz AG, Bonaduz, Switzerland) was used for selective amplification, multiplexing of PCR products and loading of the 96-well sequencer plate, and 38 individuals (9%) were independently replicated for all 21 primer combinations (see [40] for more details).

### AFLP fragment scoring and diversity

AFLP fragment scoring was performed with GeneMapper software version 3.7 (Applied Biosystems). Bin sets were created automatically and manually revised [40]. Two AFLP data matrices were produced, one with band intensity information and one with a standard binary presence-absence matrix. The AFLPs binary data matrix was used to estimate reproducibility, AFLP diversity estimates, and to run the first PCA analyses. A particular AFLP band intensity was scored as ‘present’ (1), if its value was larger than 10% of the 95% band intensity distribution quantile, or ‘absent’ (0), if its intensity was smaller than 10% of the 95% quantile value. AFLP marker frequencies, the number of variable markers per population and AFLP diversity were then calculated with the program AFLPDAT [70]. AFLP diversity was calculated as the average proportion of pairwise differences between individuals for each population, which is an index similar to Nei's gene diversity calculated from marker frequencies [71], [72].

### Outlier detection

A Bayesian genome scan approach (BayeScan) was used to detect markers under selection. This procedure is more efficient than classical outlier detection methods (like DETSELD, modified version of [73] or DFDIST, modified version of [74]) in the discovery of true selected loci, as it results in a lower number of false positives [75]. BayeScan 2.1 was specifically developed for AFLP markers. The inclusion of band intensity information makes the BayeScan analysis of dominant AFLPs almost as powerful as an analysis of the same number of codominant markers (e.g. reaching 92% of the power of a SNP data set) to detect selection (for more details see [40]). Moreover, this additional information makes it possible to infer population-specific inbreeding coefficient (*F*_IS_) from AFLP data [56]. Band intensity information required by BayeScan 2.1 was obtained from the AFLP data matrix of marker band intensity provided by GeneMapper. Since markers with a low minor allele frequency systematically bias the *F*_ST_ estimates downwards [76], only markers with band frequencies between 5% and 95% were used for subsequent analyses. This procedure prevents an artificial increase in the number of inferred outlier markers under positive selection [76]. Note that markers having band frequencies higher than 95% were still considered as polymorphic if the distribution of band intensity across all individuals was bimodal [40] and if they did not exceed three-times the 95% quantile of the band intensity distribution for that marker to avoid artefacts of the sequencing machine. These markers are probably informative to infer *F*_IS_, as they contain a high proportion of fixed and/or heterozygous individuals.

BayeScan assumes that allele frequencies within populations follow a multinomial-Dirichlet distribution [55], [77], [78] with *F*_ST_ parameters being a function of population-specific components shared among all loci and of locus-specific components shared among all populations. For a given locus, departure from neutrality is assumed when the locus-specific component is required to explain the observed pattern of diversity. BayeScan directly infers the posterior probability of each locus to be under the effect of selection by defining and comparing two alternative models: one model includes the locus-specific component, while the other excludes it [35]. The ratio of the model posterior probabilities is used to calculate then the posterior odds (PO), which measures how much more likely the model with selection is compared to the model without selection (see [40]). We used a threshold of PO>10 for a marker to be considered under selection, which refers to “strong evidence” for the alternative model (in this case the model with selection) as defined by Jeffreys [79]. For the Markov chain Monte Carlo (MCMC) algorithm we used 20 pilot runs of 5,000 iterations to adjust the proposal distribution to acceptance rates between 0.25 and 0.45 for the runs. A burn-in of 50,000 iterations was used and visually checked for convergence of the MCMC chains, followed by 50,000 iterations for estimation using a thinning interval of 10. False Discovery Rate (FDR) was used to control for multiple testing [40], [80].

### Inference of neutral genetic structure across Europe

We performed two principal component analyses (PCA) in R [81] to infer the patterns of neutral genetic structure in common voles across Europe. PCA analyses were performed on the neutral (excluding outlier loci) and evolutionary informative AFLP markers. Evolutionary informative AFLPs have band frequencies ranging between 5% and 95%, which excludes uninformative and rare markers [76]. One PCA analysis was done at the individual level using AFLP marker presence/absence data for all 444 individuals and the second analysis was done at the population level, on the basis of marker allele frequencies estimated by BayeScan [40], [56] using band intensity information.

### Inference of balancing selection

Markers detected under balancing selection were investigated in more detail, as heterozygosity information can be gained from the population-specific band intensity distribution for a specific AFLP marker. A marker under balancing selection should indeed have evenly distributed allele frequencies across most populations and heterozygous individuals should be observed within populations, leading to a bimodal band intensity distribution for this AFLP marker [56]. The markers inferred as under balancing selection were thus carefully examined for bimodality of band intensities. However, sex-chromosome linked markers may also show bimodal distributions and low differentiation between populations in samples with equal sex ratios, as males only have one X-chromosome. A t-test implemented in R was thus used to check for association between band intensity and gender, using a threshold of p>0.05 without correction for multiple testing, to be conservative in the identification of marker under balancing selection. We have used the same approach to test for any amplification difference among different 96-well PCR plates of the same primer pair (batch effect).

### Inference of positive selection patterns across Europe

To infer the patterns of positive selection in common voles across Europe we performed scaled PCA in R of the population allele frequencies of loci inferred under positive selection by BayeScan.

To identify the strongest geographic patterns of selection across Europe, we used a locus-by-locus SAMOVA approach [82] to separate for each marker populations into groups (k = 2) leading to the highest level of genetic differentiation (*F*_CT_). The three outlier loci showing the highest *F*_CT_ were identified and plotted onto the European map using the R package plotrix to visualize the population-specific allele frequencies of these patterns of selection. To find loci showing similar geographic patterns of selection across Europe, which could be the cause of multi-genic adaptation due to similar selective pressures on different loci or genetic linkage of markers, we computed a pairwise Pearson's correlation between the population-specific allele frequencies of the outlier loci using the R package psych and Holm's correction for multiple testing [83].

## Results

### AFLP variation and neutral genetic structure across Europe

The AFLP analyses of the 21 European vole populations provided 3,839 markers. The majority of these AFLP markers were polymorphic (3,318; 86%) and 2,054 (54%) showed informative band frequencies between 5% and 95% overall. For each individual, we obtained on average 2,342 AFLPs (range: 2,169–2,418) across all primer combinations, and the mean length of the fragments was 239 bp. An average of 183 AFLP markers was scored per primer combination across all individuals (range: 86–256; Table 2). The average proportion of variable AFLP bands per population was 31%, with an average AFLP diversity of 9.6%. *F*_IS_ estimates were low for all populations, ranging between 0.001 and 0.043 (Table 1). Average genetic differentiation among populations was globally high with an average population-specific *F*_ST_ of 0.31. The population from the Czech Republic (CZD) had the highest number of variable AFLP bands per population (46%), and consequently the lowest population-specific *F*_ST_ (0.16), whereas the lowest diversity was observed in a population of the Swiss Alps (CHMe), with only 20% of variable markers and hence a high population-specific *F*_ST_ (0.41).

**Table 2:** AFLP markers detected under positive selection with BayeScan.

Primers	Markers	# BayeScan	Marker ID of specific primer combination
*ACag*	201	9	*33; 77; 84; 85; 119; 136; 186; 187; 200*
*ACtc*	175	2	*21; 94*
*ACtt*	157	5	*40; 76; 79; 115; 142*
*AGaa*	147	4	*5; 56; 60; 101*
*AGac*	200	7	*3; 13; 84; 144; 161; 188; 190*
*AGtg*	141	4	*97; 103; 106; 126*
*CAat*	210	5	*102; 103; 145; 166; 181*
*CAta*	185	8	*73; 84; 116; 155; 157; 158; 165; 169*
*CCac*	195	4	*17; 129; 153; 167*
*CCta*	219	3	*88; 153; 213;*
*CCtt*	256	12	*2; 34; 41; 42; 50; 67; 97; 158; 168; 169; 193; 208*
*CGag*	101	4	*67; 69; 70; 97*
*CGtt*	86	6	*24; 40; 50; 51; 79; 87*
*CTaa*	239	11	*3; 7; 78; 86; 166; 173; 184; 185; 187; 208; 232*
*CTag*	208	9	*64; 90; 102; 125; 132; 133; 147; 154; 176*
*CTtg*	211	10	*46; 93; 97; 104; 117; 133; 170; 174; 175; 187*
*GCat*	224	12	*15; 31; 60; 106; 169; 175; 180; 181; 192; 208; 212; 213*
*GCta*	199	6	*7; 20; 33; 82; 98; 187*
*GCtc*	181	7	*16; 22; 40; 43; 160; 166; 172*
*GGac*	168	4	*30; 31; 36; 164*
*GGtc*	136	6	*15; 18; 64; 85; 125; 129*
Total	3839	138	

Given are the 21 primer combinations investigated, the total number of scored AFLP markers per primer combination across all individuals, the number of markers with a posterior odds (PO)>10 and the primer combination specific IDs of the markers under selection.

**Figure 2:**
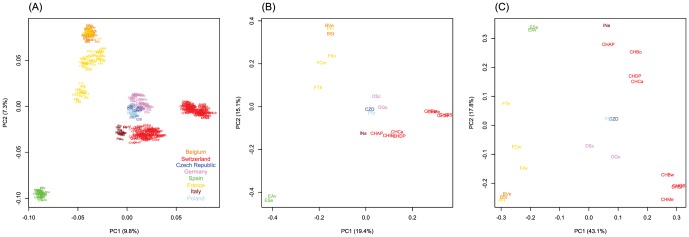
Principal component analysis (PCA) of (A) the neutral binary AFLP data matrix of 444 individuals from 21 populations across Europe using 1,843 neutral and evolutionary informative AFLP markers (see Material and Methods). (B) PCA of the 21 population-specific allele frequency estimates of neutral AFLP markers by BayeScan. The distribution of the populations on the plot roughly follows the geographic origin of the samples. (C) PCA of the estimated population-based allele frequencies of the 138 outliers probably under positive selection. For population IDs see Table 1. Colours correspond to country affiliation (see Figure 1).

The two “evolutionary neutral” PCAs were based on 1,843 neutral AFLP markers - these were the 2,054 evolutionary informative AFLP markers minus the 211 inferred outlier loci (see more details below). These neutral markers led to a clustering of individuals that approximately matches the geographic origin of the samples (Figure 1) except that the Swiss vole populations were somewhat farther apart than geography would suggest. The entire individual-based AFLP data set (Figure 2A) as well as the PCA from estimated population-specific allele frequencies (Figure 2B) show very similar patterns and allow a clear separation of the populations, which indicates the high information content of these AFLP markers.

### Genome scan

**Figure 3:**
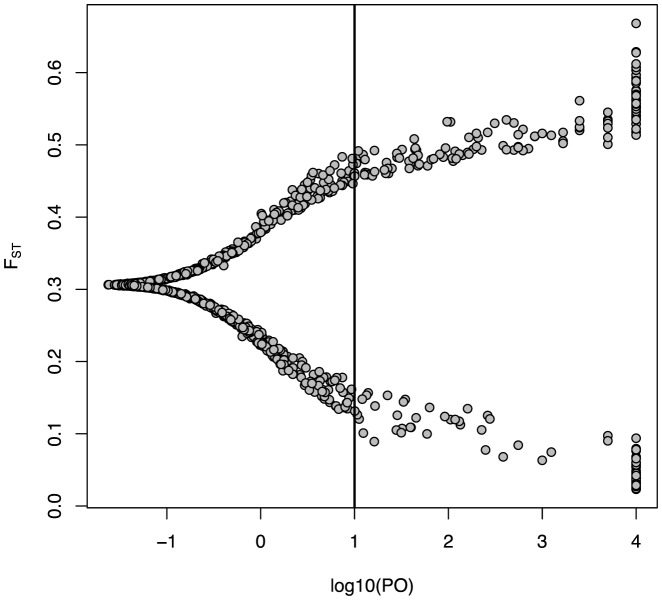
Results of BayeScan analysis for 2,054 informative AFLPs genotyped in 444 *M. arvalis* voles sampled in 21 populations. The marker-specific *F*_ST_ is plotted against the posterior odds (PO) of being under selection. The vertical line shows the critical PO of 10 used to identify outlier markers. Markers on the right side of the vertical line are outliers: 138 markers with high *F*_ST_ indicative of positive selection and 73 markers with low *F*_ST_ indicative of balancing selection were identified. Markers having a log10(PO)>4 were summarized in the category 4.

The BayeScan analysis of the 2,054 informative AFLP markers in 21 populations across Europe revealed 211 markers with a PO for selection larger than 10 with an associated FDR of less than 1.4%. Among these markers under selection, 138 (6.7%) had high *F*_ST_ (mean *F*_ST_: 0.52) indicative of positive selection, and 73 were associated with very low *F*_ST_ (mean *F*_ST_: 0.08) indicative of balancing selection (Figure 3; Table 2).

### Inference of balancing selection

**Figure 4:**
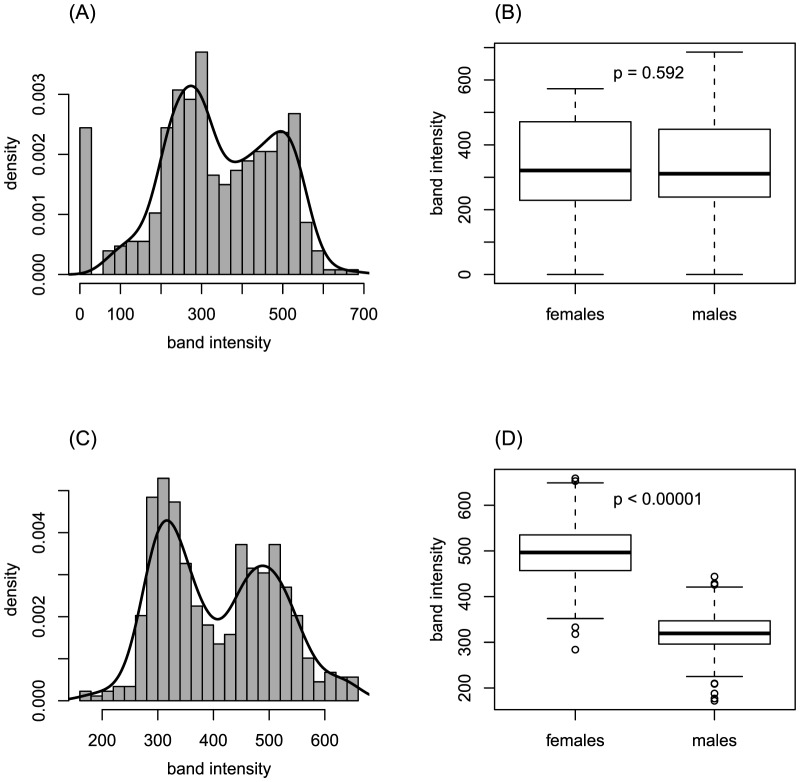
Bimodal band intensity distribution of two low *F*_ST_ outlier markers. (A) Bimodal distribution of a marker likely to be under balancing selection (*CTaa125*). The zero class of the distribution represents individuals not showing any band, the following first peak corresponds to heterozygous individuals and the second peak represents homozygous individuals. The black line represents a fitted density curve. (B) Comparison of band intensities in males and females for marker *CTaa125* shown in (A) revealing no statistical difference and suggesting it is an autosomal marker. (C) Bimodal distribution for marker *ACtt16*. (D) Corresponding box plot of sex-specific band intensities for marker *ACtt16* where females have band intensities about twice larger than males, hence suggesting it is an X-linked marker.

Bimodal band intensity information of AFLPs (for more details see Figure 4A and B, or [40], [56], [57]) was used to identify prime candidates for balancing selection and to exclude false positives among the 73 low *F*_ST_ outliers. Among these, 40 markers were considered as unlikely to be under balancing selection, either because outliers showed significant band intensity differences between males and females (t-test, p<0.05) and were thus likely sex-chromosome linked (33 markers, Table 3, Figure 4C and D) or because of PCR amplification strength differences between 96-well plates (7 markers of the primer combination *GGtc*).

**Table 3:** List of the 73 markers identified by BayeScan as being potentially under balancing selection.

			Frequency		
ID	PO	*F*_ST_	Marker	Allele	Marker categorization
*CCta185*	226.3	0.105	0.991	0.843	bimodal (balancing selection)
*CGag21*	∞	0.057	0.918	0.764	bimodal (balancing selection)
*CTaa125*	63.1	0.136	0.927	0.699	bimodal (balancing selection)
*CTaa235*	14.0	0.157	0.813	0.618	bimodal (balancing selection)
*GCat153*	∞	0.094	0.723	0.476	bimodal (balancing selection)
*GCtc42*	118.0	0.120	0.904	0.770	bimodal (balancing selection)
*CAta44*	∞	0.032	1	0.738	bimodal a
*GCtc76*	∞	0.029	0.984	0.847	bimodal a
*ACtc64*	999.0	0.063	0.995	0.972	multimodal b
*AGaa48*	554.6	0.084	1	0.844	multimodal b
*AGaa96*	22.5	0.153	0.785	0.546	unimodal b
*AGtg79*	130.6	0.117	0.941	0.728	unimodal b
*CAat185*	249.0	0.078	0.969	0.812	unimodal b
*CAta9*	∞	0.035	1	0.940	unimodal b
*CAta91*	32.1	0.107	0.932	0.783	unimodal b
*CCta61*	28.6	0.126	0.934	0.761	unimodal b
*GCat12*	∞	0.033	1	0.982	unimodal b
*GCat95*	∞	0.054	0.913	0.739	unimodal b
*GCat152*	1249	0.075	0.93	0.740	unimodal b
*GCta26*	91.6	0.124	0.888	0.696	unimodal b
*GCtc48*	12.1	0.148	0.857	0.596	unimodal b
*ACag100*	134.1	0.113	0.226	0.131	low frequency c
*CCac84*	11.2	0.120	0.052	0.049	low frequency c
*CCta81*	27.9	0.105	0.074	0.044	low frequency c
*CCta90*	59.2	0.100	0.064	0.052	low frequency c
*CCta180*	31.5	0.102	0.06	0.037	low frequency c
*CCta184*	10.7	0.127	0.065	0.043	low frequency c
*CCtt32*	10.1	0.132	0.07	0.054	low frequency c
*CCtt65*	∞	0.078	0.33	0.206	low frequency c
*CCtt131*	39.3	0.109	0.119	0.082	low frequency c
*CCtt145*	383.6	0.068	0.075	0.074	low frequency c
*CGtt4*	16.5	0.139	0.204	0.123	low frequency c
*CGtt6*	40.0	0.109	0.098	0.069	low frequency c
*ACtc47*	∞	0.066	0.991	0.812	sex-linked d
*ACtt16*	∞	0.026	1	0.787	sex-linked d
*ACtt18*	105.4	0.120	0.881	0.734	sex-linked d
*ACtt31*	∞	0.024	0.998	0.762	sex-linked d
*ACtt46*	276.8	0.121	0.938	0.713	sex-linked d
*AGac127*	∞	0.079	0.998	0.829	sex-linked d
*AGtg131*	160.3	0.135	0.78	0.596	sex-linked d
*CAat112*	33.2	0.144	0.847	0.618	sex-linked d
*CAta114*	∞	0.024	0.986	0.744	sex-linked d
*CAta142*	∞	0.068	0.961	0.772	sex-linked d
*CCac46*	∞	0.038	0.995	0.747	sex-linked d
*CCac57*	∞	0.029	1	0.763	sex-linked d
*CCta11*	∞	0.024	1	0.752	sex-linked d
*CCta29*	34.7	0.148	0.66	0.477	sex-linked d
*CCta73*	12.4	0.101	0.998	0.983	sex-linked d
*CCtt100*	∞	0.031	0.991	0.710	sex-linked d
*CCtt150*	∞	0.042	0.998	0.757	sex-linked d
*CCtt191*	∞	0.029	0.995	0.712	sex-linked d
*CTaa27*	∞	0.040	0.984	0.775	sex-linked d
*CTaa102*	∞	0.040	0.934	0.662	sex-linked d
*CTaa113*	∞	0.049	1	0.780	sex-linked d
*CTag19*	∞	0.045	1	0.772	sex-linked d
*CTag94*	∞	0.027	0.998	0.797	sex-linked d
*CTtg10*	∞	0.076	0.896	0.738	sex-linked d
*CTtg105*	∞	0.038	0.971	0.730	sex-linked d
*CTtg155*	∞	0.059	0.943	0.721	sex-linked d
*GCat44*	∞	0.068	0.946	0.778	sex-linked d
*GCat69*	∞	0.023	0.995	0.742	sex-linked d
*GCat98*	∞	0.044	0.995	0.792	sex-linked d
*GCta14*	∞	0.060	1	0.817	sex-linked d
*GCta96*	4999	0.090	0.895	0.632	sex-linked d
*ACag54*	262.2	0.125	0.576	0.831	sex-linked d
*AGtg13*	13.4	0.153	0.444	0.301	sex-linked d
*GGtc1*	∞	0.033	0.998	0.866	lab amplification difference e
*GGtc9*	45.3	0.122	0.988	0.748	lab amplification difference e
*GGtc10*	∞	0.047	0.995	0.981	lab amplification difference e
*GGtc21*	4999	0.097	0.846	0.714	lab amplification difference e
*GGtc34*	16.2	0.089	0.995	0.968	lab amplification difference e
*GGtc37*	∞	0.023	0.998	0.863	lab amplification difference e
*GGtc55*	∞	0.057	0.998	0.817	lab amplification difference e

The top six markers are prime candidates for being under balancing selection (underlined markers) due to their clear bimodal intensity distribution in all populations. Thirty-three additional markers are sex-lined. Given are marker ID, posterior odds (PO) for the marker, marker frequency, estimated allele frequency and marker categorization.

a clear bimodality was not found in single population.

b unimodal or multimodal band intensity distribution.

c low allele frequency.

d sex-chromosome linkage.

e bimodal distribution probably due to PCR amplification strength difference between 96-well plates of one specific primer pair (*GGtc*).

**Figure 5:**
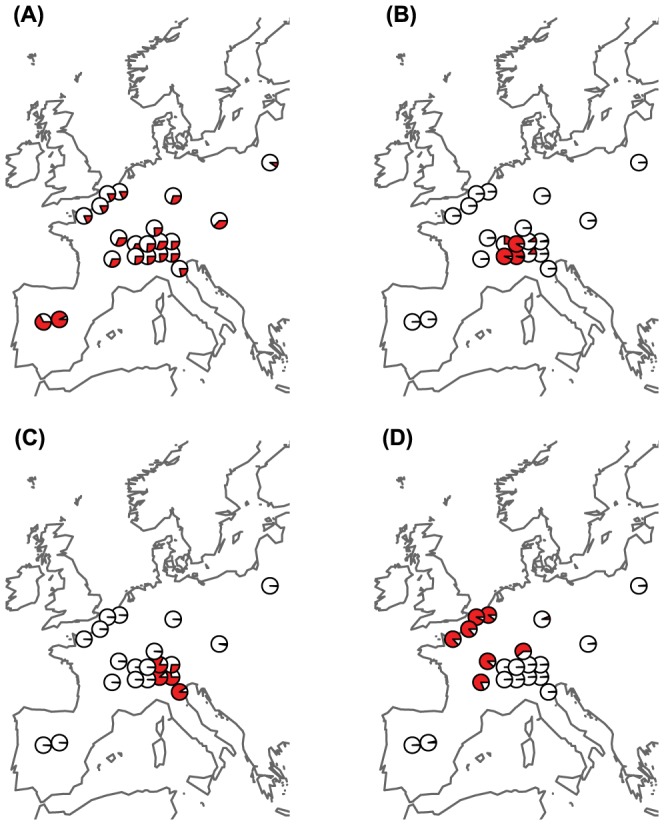
Allele frequency distributions of a locus potentially under balancing and three loci potentially under positive selection across Europe. Pie charts indicate the minor allele frequency. (A) Potential patterns of continental balancing selection of the locus *CTaa125*, which shows very homogenous allele frequencies across Europe. (B–D) Three loci under potential positive selection, which produced the strongest splits (*F*_CT_) between two groups of populations across Europe identified by the locus-by-locus SAMOVA approach [82]. Shown are the loci (B) *ACag119* with a *F*_CT_ of 0.93, (C) CTaa3 with a *F*_CT_ of 0.89, and (D) GGac31 with a *F*_CT_ of 0.87.

Among the remaining 33 markers with low *F*_ST_ values, 27 showed distributions that could be compatible with other factors than just balancing selection. Two markers (*CAta44* and *GCtc76*) had an overall bimodal distribution, but a clear bimodality was missing in individual populations. Thirteen markers had either a unimodal or multimodal band intensity distribution. Twelve markers had low allele frequencies (0.04–0.21) that could be a consequence of negative selection or frequency dependent selection, which is also form of balancing selection. Finally, six markers were identified as prime candidates for balancing selection (Table 3), as homozygous individuals had approximately twice the band intensity of heterozygous individuals (Figure 4A) and all populations showed intermediate allele frequencies across the European continent (see e.g. Figure 5A).

### Inference of positive selection across Europe

We detected a total of 138 markers potentially under positive selection across Europe, with an average of 6.6 outliers per tested primer combination (range: 2–12; Table 2). For these outliers, strong allele frequency differences were always identified in three or more populations compared to the rest, showing that selection was inferred independently in multiple populations (see e.g. Figure 5 B–D).

The PCA based on allele frequencies estimated for the 138 loci potentially under positive selection revealed a different pattern than the neutral markers (Figure 2C). Especially the populations within the Swiss Alps (CHAP, CHBo, CHCa, CHDP, CHBw, CHMe, CHGS and CHSF) and Italian Alps (INa) are much more separated from the other populations and show larger extent of differentiation among themselves compared to the PCA on neutral loci (Figure 2A and B), which is potentially indicative of strong selection pressures in the alpine area.

SAMOVA allowed us to identify the outlier loci that produced the strongest splits between two groups of populations across Europe leading to the highest level of genetic differentiation (*F*_CT_), which might be an indication of the strength of selection. The three loci that showed the strongest splits are *ACag119* with a *F*_CT_ of 0.93 (Figure 5B), *CTaa3* with a *F*_CT_ of 0.89 (Figure 5C) and *GGac31* with a *F*_CT_ of 0.87 (Figure 5D). The pairwise comparison of allele frequencies of outlier loci identified that *ACag119* showed a significant correlation with only three other loci, *CTaa3* with six and *GGac31* with 16 loci. Among the 138 loci under positive selection the average number of associations was 6.1 with a range of 0 to 24 associations. Additional information for all 138 outlier loci can be found in Table S2.

## Discussion

The current study illustrates the capacity of Bayesian *F*_ST_ outlier approaches to identify the signature of positive and balancing selection in non-model organisms. The nearly 4,000 AFLP markers, of which 2,054 were evolutionarily informative, clearly allowed us to screen a representative part of the common vole genome for loci linked to recent adaptation on a continental scale in Europe.

### Genetic structure across Europe inferred by AFLPs

The neutral AFLP markers allowed us to accurately resolve population genetic structure of the 21 vole populations across the European continent and the PCA led to a clustering of individuals and populations that corresponds approximately to the geographic origin of the samples (Figure 1 vs. 4A and B). Similar patterns were found in humans were genetic data also mirror geography in Europe [84]. This high resolution indicates the large information content present in this AFLP data set and is further supported by a very similar PCA-based clustering of populations inferred by 6,807 polymorphic SNPs (see Figure S2 in [60]), which were used to resolved the four evolutionary lineages present in Europe [43].

### Pattern of selection across European continent

We scanned 21 vole populations across the European continent for evidence of selection. Overall slightly more than 8% of all markers were under positive or balancing selection. Despite the detection of some candidate loci for balancing selection (1.6%), more loci for local positive selection (6.7%) were identified. These results suggest that drift and the demographic history of vole populations have strongly influenced the observed genetic diversity, but that also positive selection plays an important role in shaping the genetic diversity of vole populations, while balancing selection is less common. Nevertheless, the detection of several markers with multiple evidence of balancing selection is remarkable, especially the signature of a stabilizing evolutionary process on such a large geographic scale.

Contrasting to our results, balancing selection played in the past an important role in evolutionary genetics in explaining the high level of genomic polymorphism observed among species or populations [8], [10]. Six decades ago Dobzhansky [1] suggested that genetic polymorphisms were maintained in populations by selection favouring heterozygotes, thus by balancing selection. Later Kimura [2], [85] has shown that most polymorphisms in the genome should be selectively neutral after the action of purifying selection. It follows that clear examples of balancing selection in any organism should be quite limited and mainly inferred by a candidate gene approach (see Introduction and e.g. [12]–[25]), but little is known about the prevalence of balancing selection on a genome wide scale [26]–[28]. In humans balancing selection is thought to have a limited role in preserving genome-wide polymorphisms [26], [86], as a specific survey of balancing selection in humans identified only 60 out of 13,400 genes [27]. In this study we identified 33 loci with significantly low levels of differentiation among populations, which represent slightly more than 1.5% of all informative markers and hence slightly more that the 0.5% inferred in humans [27]. Our findings, together with the human studies [27], indicate that large geographic scale balancing selection is probably not as frequent as previously suspected, and hence only plays a minor role in maintaining polymorphism in a population or in shaping the genetic diversity of a species.

The observation of evenly distributed allele frequencies across the whole European continent (e.g. see Figure 5A) despite extremely strong levels of differentiation among populations (average *F*_ST_ = 0.31) is quite remarkable, especially for a species with limited dispersal ability [43], [87]. Such even allele frequencies across a large geographic range are difficult to explain in absence of strong stabilizing selection and hence good support for the presence of balancing selection.

This study used a conservative *post-hoc* evaluation of AFLP marker band intensity distributions to provide further support for the authenticity of the signature of balancing selection, which allowed us to prioritize prime candidate loci for balancing selection. Six markers were characterized by low *F*_ST_ values, evenly distributed allele frequencies among populations (Figure 5A) and especially by the bimodal band intensity distribution, which clearly indicates the presence of heterozygous individuals in several populations (Figure 4A). Apart from these six loci, 27 markers showed peculiarities also compatible with other factors than only balancing selection. Twelve markers had low allele frequencies across Europe, maybe as a result of frequency-dependent selection, a selective mechanism that favours alleles when they are rare and might result in balanced genetic polymorphisms in populations [11]. But the observed low allele frequencies might also be explained by slightly negative selection [27]. For 15 markers no obvious bimodal band intensity distribution was observed, hence no clear signal of heterozygous individuals within populations could be identified, which might be explained by the stochasticity of slight technical variation in the sequencing machine that might have eroded the signal. However, especially the detection of 33 sex-chromosome linked markers (Figure 4B) clearly supports the use of AFLPs as a partially codominant marker system and indicates that heterozygous individuals or individuals carrying only one gene copy can reliably be estimated from the band intensity distribution in AFLP markers [56], [57].

Compared to balancing selection the inference of directional selection is less questioned, even though some confounding demographic factors (e.g. surfing during range expansions; [88], [89]) might produce some false positives. However, as we have used a quite stringent threshold for accepting a locus to be under selection (PO>10), our results suggest that we have here a very low false discovery rate of less than 1.4%. We detected that 6.7% of the informative markers probably evolved as a consequence of directional selection, which might be linked to adaptation to spatial heterogeneity of the environments of European vole populations. Given the wide distribution range and highly heterogeneous environments where these voles are found, it is indeed expected that different polymorphisms might be selected in different populations and habitats [5], [26]. The markers detected under positive selection in this study display a wide variety of allele frequency patterns across Europe. The PCA based on 138 markers under positive selection revealed a quite different structure (Figure 2C) than the PCA computed on 1,843 informative and neutral AFLPs (Figure 2A and B), indicating that selection acts differently on these loci than the interplay of drift and geographic separation. It is difficult to draw conclusions on the selection pressure from the allele frequency distribution of these markers; nevertheless there are some interesting patterns, which might be explained by environmental differences among populations. The two outlier loci that showed the strongest splits between two groups of populations across Europe (Figure 5B and C), were driven by populations from Alpine areas (some of the vole populations lived above 2,000 m asl). Hence they might be related to an adaptation to high elevation [40], [90] or just to the highly heterogeneous environment observed at a small geographic scale, which is specific to Alpine regions [91]. These Swiss and Italian Alpine populations are much more separated in the PCA on loci under selection (Figure 2A) than in the neutral PCAs, indicating that probably many loci are under selection in this region. However, there are also patterns that are more difficult to interpret in environmental or geographic context, e.g. Figure 5D, but biotic interactions can be also very important for local adaption and are much more difficult to infer.

### Outlook

AFLP genome scans enable us to detect markers under recent selection in the common vole genome, but it is unfortunately impossible to determine their function and location in the absence of a sequenced genome for this species. New high-throughput technologies make full genomes more accessible than before (for review see [92]–[94]), but target-capture sequencing of hundreds of individuals is still prohibitive for most non-model organisms [57] and full genome re-sequencing studies of pooled population data (Pool-Seq) is only possible for rather small genomes (see e.g. [91], [95], [96]). An alternative strategy would be the investigation of candidate loci for selection by direct high-throughput sequencing of AFLP fragments [60], [97], which could be useful to further characterize candidate regions and genes linked with AFLP markers in this non-model organism.

## Supporting Information

Table S1 The 21 selective primer combinations and their fluorescent labels used in the AFLP assay.
(DOCX)

Table S2 SAMOVA results of the 138 loci probably under positive selection ranked by *F*_CT_ and the number of significant association with other loci having similar allele frequencies across Europe.
(DOCX)
