# Supplementary material for: Continental-Scale Footprint of Balancing and Positive Selection in a Small Rodent (Microtus arvalis)
Source: PLoS One. 2014 Nov 10;9(11):e112332. doi: 10.1371/journal.pone.0112332 (PMC4226552; doi:10.1371/journal.pone.0112332)
Supplement: Table S1 — The 21 selective primer combinations and their fluorescent labels used in the AFLP assay. (DOCX) [file pone.0112332.s001.docx]

**Table S1** The 21 selective primer combinations (marked with X) and their fluorescent labels used in the AFLP assay^a^.

|  | ***Mse*I** |  |  |  |  |  |  |  |
| --- | --- | --- | --- | --- | --- | --- | --- | --- |
| ***Eco***RI | CAA | CAC | CAG | CAT | CTA | CTC | CTG | CTT |
| AAC, NED |  |  | X |  |  | X |  | X |
| AAG, NED | X | X |  |  |  |  | X |  |
| ACA,6-FAM |  |  |  | X | X |  |  |  |
| ACC, VIC |  | X |  |  | X |  |  | X |
| ACG, PET |  |  | X |  |  |  |  | X |
| ACT, 6-FAM | X |  | X |  |  |  | X |  |
| AGC, VIC |  |  |  | X | X | X |  |  |
| AGG, PET |  | X |  |  |  | X |  |  |

^a^Rows contain selective tri-nucleotide extensions attached to the 3’ end of the fluorescently labeled *Eco*RI primer 5’-GACTGCGTACCAATTC–NNN-3’. Columns contain selective tri-nucleotide extensions attached to the 3’ end of the *Mse*I primer 5’-GATGAGTCCTGACCGA–NNN-3’.
