# Supplementary material for: Continental-Scale Footprint of Balancing and Positive Selection in a Small Rodent (Microtus arvalis)
Source: PLoS One. 2014 Nov 10;9(11):e112332. doi: 10.1371/journal.pone.0112332 (PMC4226552; doi:10.1371/journal.pone.0112332)
Supplement: Table S2 — SAMOVA results of the 138 loci probably under positive selection ranked by F CT and the number of significant association with other loci having similar allele frequencies across Europe. (DOCX) [file pone.0112332.s002.docx]

**Table S2** SAMOVA results of the 138 loci probably under positive selection ranked by the *F*_CT_ and number of significant association with other loci having similar allele frequencies across Europe.

| Locus | #pops/ group1 | Pops in group 1 | #pops/ group2 | Pops in group 2 | Fct | #associations |
| --- | --- | --- | --- | --- | --- | --- |
| *ACag119* | 3 | 8 9 10 | 18 | 1 2 3 4 5 6 7 11 12 13 14 15 16 17 18 19 20 21 | 0.92634 | 3 |
| *CTaa3* | 17 | 1 2 5 6 8 9 10 11 12 13 14 15 16 17 18 19 21 | 4 | 3 4 7 20 | 0.88718 | 5 |
| *GGac31* | 7 | 1 2 13 16 17 18 19 | 14 | 3 4 5 6 7 8 9 10 11 12 14 15 20 21 | 0.87281 | 1 |
| *CAta84* | 18 | 1 2 3 4 6 7 9 11 12 13 14 15 16 17 18 19 20 21 | 3 | 5 8 10 | 0.86304 | 1 |
| *GCat180* | 17 | 1 2 5 6 8 9 10 11 12 13 14 15 16 17 18 19 21 | 4 | 3 4 7 20 | 0.85962 | 13 |
| *GGac36* | 17 | 3 4 5 6 7 8 9 10 11 12 13 14 15 17 19 20 21 | 4 | 1 2 16 18 | 0.85949 | 0 |
| *GCta7* | 6 | 1 2 16 17 18 19 | 15 | 3 4 5 6 7 8 9 10 11 12 13 14 15 20 21 | 0.85603 | 1 |
| *CTaa185* | 18 | 1 2 3 4 5 6 7 8 9 10 11 12 13 16 17 18 20 21 | 3 | 14 15 19 | 0.84305 | 1 |
| *CGag97* | 3 | 1 2 18 | 18 | 3 4 5 6 7 8 9 10 11 12 13 14 15 16 17 19 20 21 | 0.83426 | 0 |
| *AGaa56* | 5 | 3 4 6 7 20 | 16 | 1 2 5 8 9 10 11 12 13 14 15 16 17 18 19 21 | 0.8335 | 0 |
| *ACtc21* | 17 | 1 2 3 4 6 7 11 12 13 14 15 16 17 18 19 20 21 | 4 | 5 8 9 10 | 0.8246 | 2 |
| *GCtc166* | 6 | 1 2 16 17 18 19 | 15 | 3 4 5 6 7 8 9 10 11 12 13 14 15 20 21 | 0.81894 | 1 |
| *GGtc125* | 4 | 1 2 16 18 | 17 | 3 4 5 6 7 8 9 10 11 12 13 14 15 17 19 20 21 | 0.81722 | 1 |
| *CAat181* | 18 | 1 2 3 4 5 6 7 8 9 10 11 12 13 16 17 18 20 21 | 3 | 14 15 19 | 0.81666 | 1 |
| *AGaa5* | 18 | 1 2 3 4 5 6 7 8 9 10 11 12 13 16 17 18 20 21 | 3 | 14 15 19 | 0.80622 | 0 |
| *GCtc22* | 6 | 1 2 16 17 18 19 | 15 | 3 4 5 6 7 8 9 10 11 12 13 14 15 20 21 | 0.80133 | 2 |
| *GCat31* | 4 | 3 4 7 20 | 17 | 1 2 5 6 8 9 10 11 12 13 14 15 16 17 18 19 21 | 0.80076 | 6 |
| *AGtg126* | 2 | 11 21 | 19 | 1 2 3 4 5 6 7 8 9 10 12 13 14 15 16 17 18 19 20 | 0.79817 | 0 |
| *GCta20* | 3 | 14 15 19 | 18 | 1 2 3 4 5 6 7 8 9 10 11 12 13 16 17 18 20 21 | 0.7927 | 2 |
| *CCac153* | 3 | 8 9 10 | 18 | 1 2 3 4 5 6 7 11 12 13 14 15 16 17 18 19 20 21 | 0.79124 | 5 |
| *CGtt51* | 4 | 14 15 16 17 | 17 | 1 2 3 4 5 6 7 8 9 10 11 12 13 18 19 20 21 | 0.78994 | 6 |
| *GGtc18* | 3 | 1 2 18 | 18 | 3 4 5 6 7 8 9 10 11 12 13 14 15 16 17 19 20 21 | 0.78671 | 1 |
| *CTag90* | 5 | 3 4 6 20 21 | 16 | 1 2 5 7 8 9 10 11 12 13 14 15 16 17 18 19 | 0.7836 | 1 |
| *CTtg97* | 7 | 1 14 15 16 17 18 19 | 14 | 2 3 4 5 6 7 8 9 10 11 12 13 20 21 | 0.77163 | 2 |
| *GGtc85* | 3 | 14 15 19 | 18 | 1 2 3 4 5 6 7 8 9 10 11 12 13 16 17 18 20 21 | 0.75807 | 6 |
| *CTtg187* | 4 | 1 2 18 19 | 17 | 3 4 5 6 7 8 9 10 11 12 13 14 15 16 17 20 21 | 0.75759 | 17 |
| *CTaa187* | 4 | 13 14 15 19 | 17 | 1 2 3 4 5 6 7 8 9 10 11 12 16 17 18 20 21 | 0.75348 | 2 |
| *GGtc64* | 16 | 3 4 5 6 7 8 9 10 11 12 13 14 15 19 20 21 | 5 | 1 2 16 17 18 | 0.75331 | 1 |
| *GCat213* | 4 | 1 2 18 19 | 17 | 3 4 5 6 7 8 9 10 11 12 13 14 15 16 17 20 21 | 0.75074 | 0 |
| *GCat208* | 5 | 5 8 9 10 21 | 16 | 1 2 3 4 6 7 11 12 13 14 15 16 17 18 19 20 | 0.74977 | 0 |
| *CCtt41* | 12 | 1 2 3 6 7 13 14 15 16 17 18 19 | 9 | 4 5 8 9 10 11 12 20 21 | 0.7436 | 2 |
| *CAat145* | 5 | 3 4 6 7 20 | 16 | 1 2 5 8 9 10 11 12 13 14 15 16 17 18 19 21 | 0.74322 | 0 |
| *AGac84* | 17 | 3 4 5 6 7 8 9 10 11 12 13 14 15 16 19 20 21 | 4 | 1 2 17 18 | 0.74247 | 3 |
| *GCat15* | 5 | 1 2 16 17 18 | 16 | 3 4 5 6 7 8 9 10 11 12 13 14 15 19 20 21 | 0.74207 | 9 |
| *ACtt40* | 17 | 1 2 3 5 6 7 8 9 10 11 12 13 16 17 18 19 21 | 4 | 4 14 15 20 | 0.742 | 1 |
| *ACag136* | 16 | 1 2 3 4 6 7 11 13 14 15 16 17 18 19 20 21 | 5 | 5 8 9 10 12 | 0.73587 | 5 |
| *CCtt97* | 7 | 2 14 15 16 17 18 19 | 14 | 1 3 4 5 6 7 8 9 10 11 12 13 20 21 | 0.73404 | 4 |
| *AGac190* | 4 | 14 15 19 21 | 17 | 1 2 3 4 5 6 7 8 9 10 11 12 13 16 17 18 20 | 0.73397 | 6 |
| *CTag154* | 17 | 1 2 4 5 6 7 8 9 10 11 12 13 16 17 18 19 21 | 4 | 3 14 15 20 | 0.73262 | 3 |
| *GCat192* | 8 | 3 4 6 7 8 9 10 20 | 13 | 1 2 5 11 12 13 14 15 16 17 18 19 21 | 0.72963 | 0 |
| *CTaa232* | 16 | 2 3 4 5 6 7 8 9 10 11 12 13 14 15 20 21 | 5 | 1 16 17 18 19 | 0.72653 | 1 |
| *AGac3* | 9 | 4 5 6 7 8 10 11 12 21 | 12 | 1 2 3 9 13 14 15 16 17 18 19 20 | 0.72505 | 23 |
| *CAta155* | 6 | 1 2 13 16 17 18 | 15 | 3 4 5 6 7 8 9 10 11 12 14 15 19 20 21 | 0.71602 | 1 |
| *CTaa208* | 4 | 3 4 14 15 | 17 | 1 2 5 6 7 8 9 10 11 12 13 16 17 18 19 20 21 | 0.71289 | 2 |
| *CTaa184* | 4 | 1 2 17 18 | 17 | 3 4 5 6 7 8 9 10 11 12 13 14 15 16 19 20 21 | 0.70357 | 19 |
| *CTaa7* | 3 | 16 17 18 | 18 | 1 2 3 4 5 6 7 8 9 10 11 12 13 14 15 19 20 21 | 0.69922 | 3 |
| *CAta157* | 3 | 14 15 19 | 18 | 1 2 3 4 5 6 7 8 9 10 11 12 13 16 17 18 20 21 | 0.69785 | 3 |
| *CAat102* | 5 | 5 8 9 10 16 | 16 | 1 2 3 4 6 7 11 12 13 14 15 17 18 19 20 21 | 0.69564 | 5 |
| *CTag64* | 8 | 1 2 14 15 16 17 18 19 | 13 | 3 4 5 6 7 8 9 10 11 12 13 20 21 | 0.69527 | 1 |
| *CCtt208* | 11 | 1 2 3 11 14 15 16 17 18 19 20 | 10 | 4 5 6 7 8 9 10 12 13 21 | 0.69191 | 4 |
| *AGtg97* | 9 | 1 2 14 15 16 17 18 19 21 | 12 | 3 4 5 6 7 8 9 10 11 12 13 20 | 0.67998 | 1 |
| *CTaa78* | 17 | 1 2 3 4 6 7 11 12 13 14 15 16 17 18 19 20 21 | 4 | 5 8 9 10 | 0.6797 | 2 |
| *CCtt2* | 7 | 3 6 14 15 17 19 21 | 14 | 1 2 4 5 7 8 9 10 11 12 13 16 18 20 | 0.67632 | 3 |
| *AGac188* | 6 | 2 5 8 9 10 18 | 15 | 1 3 4 6 7 11 12 13 14 15 16 17 19 20 21 | 0.67608 | 17 |
| *GCtc172* | 12 | 3 4 5 6 7 8 9 10 11 12 20 21 | 9 | 1 2 13 14 15 16 17 18 19 | 0.67519 | 1 |
| *GCat212* | 6 | 3 4 6 7 10 20 | 15 | 1 2 5 8 9 11 12 13 14 15 16 17 18 19 21 | 0.67463 | 4 |
| *CTag102* | 19 | 1 2 3 4 5 6 7 8 9 10 11 12 13 16 17 18 19 20 21 | 2 | 14 15 | 0.67205 | 0 |
| *GGtc129* | 15 | 3 4 5 6 7 8 9 10 11 12 13 14 15 20 21 | 6 | 1 2 16 17 18 19 | 0.67116 | 1 |
| *CTaa166* | 4 | 1 2 18 19 | 17 | 3 4 5 6 7 8 9 10 11 12 13 14 15 16 17 20 21 | 0.6695 | 2 |
| *CCtt34* | 12 | 3 4 5 6 7 8 9 10 11 13 20 21 | 9 | 1 2 12 14 15 16 17 18 19 | 0.66884 | 16 |
| *CAta169* | 12 | 4 5 6 7 8 9 10 11 12 13 17 21 | 9 | 1 2 3 14 15 16 18 19 20 | 0.6673 | 6 |
| *ACag186* | 12 | 3 4 5 6 7 8 9 10 11 12 20 21 | 9 | 1 2 13 14 15 16 17 18 19 | 0.6635 | 5 |
| *AGaa60* | 8 | 3 4 5 8 9 10 16 20 | 13 | 1 2 6 7 11 12 13 14 15 17 18 19 21 | 0.66085 | 6 |
| *CTtg46* | 15 | 3 4 5 6 7 8 9 10 11 12 13 16 18 20 21 | 6 | 1 2 14 15 17 19 | 0.65737 | 11 |
| *GCtc16* | 12 | 3 4 5 6 7 8 9 10 11 12 13 21 | 9 | 1 2 14 15 16 17 18 19 20 | 0.65548 | 0 |
| *CCta88* | 9 | 1 2 8 14 15 16 17 18 19 | 12 | 3 4 5 6 7 9 10 11 12 13 20 21 | 0.65333 | 1 |
| *ACag33* | 9 | 1 2 13 14 15 16 17 18 19 | 12 | 3 4 5 6 7 8 9 10 11 12 20 21 | 0.65051 | 2 |
| *CCtt168* | 11 | 1 2 5 7 8 9 10 13 16 17 18 | 10 | 3 4 6 11 12 14 15 19 20 21 | 0.64858 | 8 |
| *CGag70* | 7 | 5 8 9 10 12 14 15 | 14 | 1 2 3 4 6 7 11 13 16 17 18 19 20 21 | 0.6474 | 0 |
| *CTtg104* | 9 | 1 2 6 11 12 13 16 18 21 | 12 | 3 4 5 7 8 9 10 14 15 17 19 20 | 0.64733 | 2 |
| *CCtt193* | 6 | 3 4 6 7 9 20 | 15 | 1 2 5 8 10 11 12 13 14 15 16 17 18 19 21 | 0.64646 | 0 |
| *CTtg174* | 8 | 5 8 9 10 12 13 16 17 | 13 | 1 2 3 4 6 7 11 14 15 18 19 20 21 | 0.63783 | 21 |
| *CAta73* | 7 | 5 6 8 9 10 14 15 | 14 | 1 2 3 4 7 11 12 13 16 17 18 19 20 21 | 0.63778 | 9 |
| *ACtt76* | 16 | 1 2 3 6 7 8 9 10 11 12 13 14 16 17 18 19 | 5 | 4 5 15 20 21 | 0.63169 | 19 |
| *ACag187* | 10 | 1 2 12 13 14 15 16 17 18 19 | 11 | 3 4 5 6 7 8 9 10 11 20 21 | 0.63064 | 12 |
| *CAat103* | 8 | 3 5 7 8 9 10 11 13 | 13 | 1 2 4 6 12 14 15 16 17 18 19 20 21 | 0.62897 | 0 |
| *CCtt67* | 6 | 5 6 8 9 10 20 | 15 | 1 2 3 4 7 11 12 13 14 15 16 17 18 19 21 | 0.62624 | 10 |
| *CAta165* | 9 | 4 5 6 7 8 9 10 12 13 | 12 | 1 2 3 11 14 15 16 17 18 19 20 21 | 0.6225 | 5 |
| *CCtt158* | 11 | 4 5 6 7 8 9 10 11 12 13 21 | 10 | 1 2 3 14 15 16 17 18 19 20 | 0.61946 | 11 |
| *AGac144* | 10 | 5 8 9 10 11 12 13 14 15 21 | 11 | 1 2 3 4 6 7 16 17 18 19 20 | 0.61858 | 8 |
| *GCat181* | 7 | 3 4 7 14 15 17 20 | 14 | 1 2 5 6 8 9 10 11 12 13 16 18 19 21 | 0.61846 | 17 |
| *CTag147* | 8 | 4 5 6 7 8 9 10 16 | 13 | 1 2 3 11 12 13 14 15 17 18 19 20 21 | 0.61681 | 0 |
| *CGag67* | 12 | 1 2 3 4 7 14 15 16 17 18 19 20 | 9 | 5 6 8 9 10 11 12 13 21 | 0.6157 | 5 |
| *CGtt87* | 17 | 1 2 3 4 5 7 9 12 13 14 15 16 17 18 19 20 21 | 4 | 6 8 10 11 | 0.61505 | 24 |
| *GCat169* | 8 | 1 2 14 15 16 17 18 19 | 13 | 3 4 5 6 7 8 9 10 11 12 13 20 21 | 0.5982 | 1 |
| *CGtt50* | 6 | 5 8 9 10 12 18 | 15 | 1 2 3 4 6 7 11 13 14 15 16 17 19 20 21 | 0.59667 | 0 |
| *ACtt142* | 9 | 1 2 4 13 17 18 19 20 21 | 12 | 3 5 6 7 8 9 10 11 12 14 15 16 | 0.59624 | 3 |
| *CTag133* | 5 | 1 2 9 18 19 | 16 | 3 4 5 6 7 8 10 11 12 13 14 15 16 17 20 21 | 0.59619 | 23 |
| *GCat175* | 13 | 3 4 5 6 7 8 9 10 11 14 15 20 21 | 8 | 1 2 12 13 16 17 18 19 | 0.59538 | 11 |
| *CCta213* | 6 | 5 8 9 10 11 21 | 15 | 1 2 3 4 6 7 12 13 14 15 16 17 18 19 20 | 0.59501 | 23 |
| *CTaa86* | 16 | 1 2 3 4 6 11 12 13 14 15 16 17 18 19 20 21 | 5 | 5 7 8 9 10 | 0.59212 | 1 |
| *CTag125* | 10 | 5 8 9 10 11 12 13 14 15 21 | 11 | 1 2 3 4 6 7 16 17 18 19 20 | 0.59098 | 5 |
| *CGag69* | 11 | 1 3 4 6 14 15 16 17 18 19 20 | 10 | 2 5 7 8 9 10 11 12 13 21 | 0.59059 | 9 |
| *CTtg170* | 4 | 2 14 15 19 | 17 | 1 3 4 5 6 7 8 9 10 11 12 13 16 17 18 20 21 | 0.59018 | 15 |
| *GGac164* | 11 | 3 4 5 6 7 8 9 13 14 15 20 | 10 | 1 2 10 11 12 16 17 18 19 21 | 0.58852 | 0 |
| *CTtg93* | 13 | 1 2 5 8 10 12 13 14 15 16 17 18 19 | 8 | 3 4 6 7 9 11 20 21 | 0.58686 | 12 |
| *ACag84* | 8 | 1 2 14 15 16 17 18 19 | 13 | 3 4 5 6 7 8 9 10 11 12 13 20 21 | 0.58537 | 1 |
| *ACag77* | 11 | 1 2 5 8 9 10 11 12 13 18 21 | 10 | 3 4 6 7 14 15 16 17 19 20 | 0.58372 | 4 |
| *CAta158* | 10 | 3 4 6 7 8 10 11 13 14 15 | 11 | 1 2 5 9 12 16 17 18 19 20 21 | 0.5832 | 4 |
| *AGtg106* | 9 | 3 4 5 6 7 8 10 12 20 | 12 | 1 2 9 11 13 14 15 16 17 18 19 21 | 0.5816 | 1 |
| *CTag176* | 13 | 1 2 11 12 13 14 15 16 17 18 19 20 21 | 8 | 3 4 5 6 7 8 9 10 | 0.57582 | 1 |
| *ACag200* | 6 | 12 14 16 17 18 19 | 15 | 1 2 3 4 5 6 7 8 9 10 11 13 15 20 21 | 0.57443 | 17 |
| *CTaa173* | 10 | 1 2 12 13 14 16 17 18 19 21 | 11 | 3 4 5 6 7 8 9 10 11 15 20 | 0.57085 | 12 |
| *CTtg133* | 14 | 1 2 3 4 5 6 7 12 13 16 17 18 19 20 | 7 | 8 9 10 11 14 15 21 | 0.57034 | 1 |
| *CCac129* | 18 | 1 2 3 4 5 6 7 8 9 10 11 12 13 16 18 19 20 21 | 3 | 14 15 17 | 0.56808 | 17 |
| *AGtg103* | 5 | 5 6 8 9 10 | 16 | 1 2 3 4 7 11 12 13 14 15 16 17 18 19 20 21 | 0.56798 | 14 |
| *CCtt50* | 13 | 3 4 5 7 8 9 10 11 12 13 17 20 21 | 8 | 1 2 6 14 15 16 18 19 | 0.56228 | 4 |
| *GGtc15* | 8 | 1 2 7 11 17 18 19 21 | 13 | 3 4 5 6 8 9 10 12 13 14 15 16 20 | 0.55955 | 6 |
| *GCat60* | 8 | 2 3 5 6 8 9 10 13 | 13 | 1 4 7 11 12 14 15 16 17 18 19 20 21 | 0.5592 | 2 |
| *AGac161* | 7 | 1 2 4 12 16 18 21 | 14 | 3 5 6 7 8 9 10 11 13 14 15 17 19 20 | 0.55317 | 17 |
| *GCtc40* | 7 | 2 3 4 6 7 18 20 | 14 | 1 5 8 9 10 11 12 13 14 15 16 17 19 21 | 0.55111 | 14 |
| *CTtg175* | 14 | 2 3 4 5 6 7 8 9 10 11 12 13 19 20 | 7 | 1 14 15 16 17 18 21 | 0.55038 | 5 |
| *GGac30* | 12 | 3 4 5 6 7 8 9 10 11 12 20 21 | 9 | 1 2 13 14 15 16 17 18 19 | 0.55005 | 9 |
| *ACag85* | 8 | 3 4 5 6 7 8 10 12 | 13 | 1 2 9 11 13 14 15 16 17 18 19 20 21 | 0.54717 | 5 |
| *ACtt115* | 14 | 1 2 3 4 6 7 14 15 16 17 18 19 20 21 | 7 | 5 8 9 10 11 12 13 | 0.54322 | 17 |
| *CCac17* | 14 | 2 3 4 5 6 7 11 12 13 14 15 19 20 21 | 7 | 1 8 9 10 16 17 18 | 0.54277 | 3 |
| *ACtt79* | 12 | 4 5 8 9 10 11 13 14 15 16 17 19 | 9 | 1 2 3 6 7 12 18 20 21 | 0.53042 | 16 |
| *CTtg117* | 12 | 1 2 9 11 12 13 14 15 16 17 18 19 | 9 | 3 4 5 6 7 8 10 20 21 | 0.52634 | 8 |
| *GCta98* | 10 | 4 5 6 7 8 9 10 11 12 13 | 11 | 1 2 3 14 15 16 17 18 19 20 21 | 0.52447 | 4 |
| *AGaa101* | 13 | 1 2 4 5 6 7 8 9 10 11 12 13 18 | 8 | 3 14 15 16 17 19 20 21 | 0.52192 | 5 |
| *CAta116* | 19 | 1 2 3 4 5 8 9 10 11 12 13 14 15 16 17 18 19 20 21 | 2 | 6 7 | 0.51782 | 0 |
| *GCtc160* | 14 | 1 2 3 7 11 12 13 14 15 16 17 18 19 21 | 7 | 4 5 6 8 9 10 20 | 0.51684 | 7 |
| *CCtt169* | 11 | 3 4 5 6 8 9 10 11 12 20 21 | 10 | 1 2 7 13 14 15 16 17 18 19 | 0.51507 | 12 |
| *CAat166* | 11 | 2 3 4 5 6 7 11 12 18 20 21 | 10 | 1 8 9 10 13 14 15 16 17 19 | 0.51083 | 8 |
| *CCac167* | 12 | 1 2 4 5 6 7 8 9 10 12 13 18 | 9 | 3 11 14 15 16 17 19 20 21 | 0.50844 | 8 |
| *CCta153* | 18 | 1 2 3 4 6 7 8 10 11 12 13 14 15 16 17 18 19 21 | 3 | 5 9 20 | 0.50382 | 15 |
| *CTag132* | 7 | 1 9 12 16 17 18 19 | 14 | 2 3 4 5 6 7 8 10 11 13 14 15 20 21 | 0.50306 | 17 |
| *ACtc94* | 11 | 3 11 12 13 14 15 16 17 19 20 21 | 10 | 1 2 4 5 6 7 8 9 10 18 | 0.50091 | 4 |
| *GCat106* | 10 | 1 2 3 6 7 13 17 18 19 20 | 11 | 4 5 8 9 10 11 12 14 15 16 21 | 0.49211 | 7 |
| *GCtc43* | 16 | 3 4 5 6 7 8 9 10 11 12 13 14 15 19 20 21 | 5 | 1 2 16 17 18 | 0.48552 | 3 |
| *GCta187* | 14 | 1 2 5 6 8 9 10 11 12 13 16 17 18 19 | 7 | 3 4 7 14 15 20 21 | 0.47875 | 6 |
| *GCta82* | 17 | 1 2 3 4 5 6 7 9 11 12 13 16 17 18 19 20 21 | 4 | 8 10 14 15 | 0.46045 | 15 |
| *AGac13* | 17 | 3 4 5 6 7 8 9 10 11 12 13 14 15 17 19 20 21 | 4 | 1 2 16 18 | 0.4372 | 10 |
| *CCtt42* | 14 | 1 2 3 6 7 13 14 15 16 17 18 19 20 21 | 7 | 4 5 8 9 10 11 12 | 0.43283 | 7 |
| *GCta33* | 14 | 1 2 3 4 6 7 13 14 15 16 17 18 19 20 | 7 | 5 8 9 10 11 12 21 | 0.43001 | 1 |
| *CGtt40* | 12 | 1 2 4 11 13 14 15 16 17 18 19 20 | 9 | 3 5 6 7 8 9 10 12 21 | 0.42806 | 16 |
| *CGtt79* | 5 | 1 2 14 15 18 | 16 | 3 4 5 6 7 8 9 10 11 12 13 16 17 19 20 21 | 0.36323 | 6 |
| *CGtt24* | 12 | 1 2 5 12 13 14 15 16 17 18 19 21 | 9 | 3 4 6 7 8 9 10 11 20 | 0.35556 | 3 |

Population ID affiliation listed in group 1 and 2:

1 BSt

2 BVe

3 CHAP

4 CHBo

5 CHBw

6 CHCa

7 CHDP

8 CHGS

9 CHMe

10 CHSF

11 CZD

12 DGo

13 DSc

14 EAv

15 ESe

16 FAv

17 FCm

18 FFr

19 FTh

20 INa

21 PSr
